# Effects of *MCF2L2, ADIPOQ *and *SOX2 *genetic polymorphisms on the development of nephropathy in type 1 Diabetes Mellitus

**DOI:** 10.1186/1471-2350-11-116

**Published:** 2010-07-28

**Authors:** Dongying Zhang, Suad Efendic, Kerstin Brismar, Harvest F Gu

**Affiliations:** 1 Rolf Luft Center for Diabetes Research, Department of Molecular Medicine and Surgery, Karolinska Institutet, Karolinska University Hospital, Solna, Stockholm, Sweden

## Abstract

**Background:**

*MCF2L2, ADIPOQ *and *SOX2 *genes are located in chromosome 3q26-27, which is linked to diabetic nephropathy (DN). *ADIPOQ *and *SOX2 *genetic polymorphisms are found to be associated with DN. In the present study, we first investigated the association between *MCF2L2 *and DN, and then evaluated effects of these three genes on the development of DN.

**Methods:**

A total of 1177 type 1 diabetes patients with and without DN from the GoKinD study were genotyped with TaqMan allelic discrimination. All subjects were of European descent.

**Results:**

Leu359Ile T/G variant in the *MCF2L2 *gene was found to be associated with DN in female subjects (P = 0.017, OR = 0.701, 95%CI 0.524-0.938) but not in males. The GG genotype carriers among female patients with DN had tendency decreased creatinine and cystatin levels compared to the carriers with either TT or TG genotypes. This polymorphism *MCF2L2-*rs7639705 together with SNPs of *ADIPOQ*-rs266729 and *SOX2*-rs11915160 had combined effects on decreased risk of DN in females (P = 0.001).

**Conclusion:**

The present study provides evidence that *MCF2L2*, *ADIPOQ *and *SOX2 *genetic polymorphisms have effects on the resistance of DN in female T1D patients, and suggests that the linkage with DN in chromosome 3q may be explained by the cumulated genetic effects.

## Background

The global increase in diabetes prevalence has been accompanied by a rise in a number of patients with diabetic nephropathy (DN). This complication affects 30-40% of the patients with type 1 (T1DM) and type 2 diabetes (T2DM), and has become the most common single cause of end-stage renal disease (ESRD) [1,2]. Evidence has demonstrated that DN has genetic components [3,4]. Therefore, identification of susceptibility or resistance genes in DN will provide better knowledge of pathomechanism and future therapies in this disease [5,6].

With the approaches of genome wide scan and linkage analyses, researchers have searched for the linkage to DN along chromosomes. The linkage to DN in chromosome 3q has been evidenced repeatedly in many ethnic groups, and indicated that there reside the susceptibility and/or resistant genes for DN in this chromosomal arm [7-13]. The adiponectin (*ADIPOQ*) gene is located in chromosome 3q27. Vionnet et al. have analyzed 14 genes including the *ADIPOQ *gene selected from chromosome 3q and found that a promoter polymorphism rs17300539 (-11391A/G) in the *ADIPOQ *gene is associated with DN in Danish T1DM patients [14]. We have searched for the susceptibility and/or resistance genes for DN in chromosome 3q with a positional candidate SNP genotyping approach. Our data indicate that the *ADIPOQ *promoter polymorphism rs266729 (-11377C/G) is associated with DN in female T1DM patients [15]. Another gene encoded by sex-determining region Y-box 2 (*SOX2*) is located in chromosome 3q26.3. There is unique tag SNP rs11915160 in UTR-3' in this intron-less gene. We have recently found that this polymorphism is associated with DN in female subjects with T1DM [16].

MCF.2 cell line derived transforming sequence-like 2 (MCF2L2) is a guanine-nucleotide exchange factor (GEF) from Rho family. Evidence has demonstrated that GEFs from Rho family are signaling molecules responsible for Rho protein activity. Over-activation of Rho protein is a common component involved in the pathogenesis of diabetes and several cardiovascular disorders, including hypertension, coronary and cerebral vasospasm and atherosclerosis [17,18]. Takeuchi et al. have demonstrated that *MCF2L2 *genetic polymorphisms confer the risk susceptibility to T2DM [19]. The *MCF2L2 *gene is located in chromosome 3q27, and resides between locations of the *ADIPOQ *and *SOX2 *genes. In the present study, we first genotyped genetic polymorphisms of the *MCF2L2 *gene and then performed multiplex gene association analysis for *MCF2L2*, *ADIPOQ *and *SOX2 *to evaluate their genetic effects on the development of DN. The data may provide useful information to better understand the linkage in chromosome 3q with DN.

## Methods

### Subjects

The subjects in Genetics of Kidney Diseases in Diabetes (GoKinD) study were collected by the Juvenile Diabetes Research Foundation in collaboration with the Joslin Diabetes Centre, George Washington University, and the United States Centres for Diabetes Control and Prevention [20]. The GoKinD collection consists of both singletons and trios, however, only singletons were included in the present study. In this GoKinD cohort, ~8% of total subjects were Americans of Black, Asian, Hispanic or Indian descents. In order to avoid the error caused by the subjects from different races, this small proportion of subjects were excluded from analyses. Subsequently, a total of 599 (female 357/male 242) T1DM patients without DN and 578 (265/313) T1DM patients with DN were included, and all subjects were of European descent. Presence of DN was defined either by persistent proteinuria (urinary albumin excretion rate >300 mg/24 h) in two out of three consecutive measurements (at least one month apart), or ESRD (not due to condition other than diabetes). Absence of DN was considered as persistent normal albuminuria (< 30 mg/24 h) despite duration of T1DM for at least 15 years. Clinical parameters of all subjects were collected by the GoKinD study [20] and are represented in Table 1. Sample collection of GoKinD study was approved by the local ethics committees. Data and material transfer agreement was completed prior to the study.

**Table 1:** Clinical material

	T1DM without DN			T1DM with DN		
	**All**	**Female**	**Male**	**All**	**Female**	**Male**

N	599	357	242	578	265	313
Age (years)	40 ± 8	40 ± 9	40 ± 8	44 ± 6	44 ± 7	45 ± 6
Duration of diabetes (years)	26 ± 8	26 ± 8	26 ± 8	32 ± 8	32 ± 8	32 ± 8
HbA1c (%)	7.5 ± 1.1	7.5 ± 1.1	7.4 ± 1.1	7.4 ± 1.9	7.4 ± 2.1	7.4 ± 1.7
BMI (kg/m^2^)	26.0 ± 4.4	25.7 ± 4.7	26.6 ± 3.7	25.7 ± 5.3	25.2 ± 5.7	26.2 ± 4.8
Creatinine (mg/dL)	0.9 ± 0.2	0.8 ± 0.1	1.0 ± 0.1	2.1 ± 1.9	1.9 ± 1.6	2.3 ± 2.0
Cystatin (mg/L)	0.8 ± 0.1	0.8 ± 0.1	0.8 ± 0.1	2.2 ± 1.7	2.2 ± 1.6	2.4 ± 1.7
Cholesterol (mg/dL)	185.0 ± 31.3	188.7 ± 30.9	179.7 ± 31.2	186.3 ± 45.9	190.3 ± 47.3	183.1 ± 44.5
HDL (mg/dL)	59.1 ± 15.9	64.8 ± 15.5	51.0 ± 12.4	53.6 ± 17.3	59.3 ± 18.2	48.9 ± 15.0
Systolic BP (mm Hg)	118 ± 12	116 ± 12	122 ± 12	131 ± 19	130 ± 20	133 ± 18
Diastolic BP (mm Hg)	71 ± 8	70 ± 7	74 ± 8	74 ± 11	72 ± 11	75 ± 11

All data are means ± SD. T1DM = Type 1 diabetic patients, DN = diabetic nephropathy.

### Marker selection and genotyping

Examined SNPs in the *MCF2L2 *gene were selected based upon the information from International HapMap Project, dbSNP databases http://www.ncbi.nlm.nih.gov/SNP/. Haploview (ver4.1) was employed to visualize linkage disequilibrium (LD) and haplotype block structures (r^2 ^> 0.8, LOD threshold 3.0). Three SNPs i.e. rs684846, rs35069869 and rs35368790 in the *MCF2L2 *gene are reported to be associated with T2DM in a Japanese population [19]. Latest information from dbSNP database indicates that SNP rs684846 resides at 3'-flanking sequence but not within the *MCF2L2 *gene region. Therefore, this polymorphism was excluded from our study.

Rs7639705 is a non-synonymous SNP and causes amino acid change from Leu to Ile at the position 359 of *MCF2L2 *cDNA sequence. This polymorphism is a tag marker for LD block 8 (8 kb) in the population of European Caucasians (CEU, Haploview ver4.1). In the Japanese study, however, SNP rs7639705 was not included. In the present study, we validated this polymorphism with 32 subjects (64 chromosomes) randomly selected from the GoKinD study cohort, and then included it for genotyping analysis.

Genotyping experiments were performed with TaqMan allelic discrimination technique. SNP genotyping assays that incorporate minor groove binding (MGB) probes were purchased from Applied Biosystems (ABI 7300, Foster City, USA). The assay ID numbers and PCR protocol are available either on request or from ABI database. For quality control, the subjects (cases and controls) were distributed randomly on each PCR plate. The negative (Universal-mixture blanks) controls were included on each plate. Genotyping experiments in 20-25% of samples were performed in duplicate, and the duplication accuracy was calculated to be 99%.

### Gene-gene interaction analyses

Generalized multifactor dimensionality reduction (GMDR) is a nonparametric and genetic model-free alternative to linear or logistic regression program for detection of gene-gene or gene-environmental interactions [21,22]. We employed this program to investigate gene-gene interactions concerning *MCF2L2*-rs7639705, *AdipoQ*-rs266729 and *Sox2*-rs11915160. This program provides a number of parameters including cross-validation consistency, testing balanced accuracy and empirical P-values to assess each selected interaction. The GMDR analyses were performed separately in female and male subjects. Logistic regression models were used for confirmation of the data from GMDR analyses.

### Statistical analyses

Hardy-Weinberg equilibrium (HWE) for individual loci was assessed using the Pearson Chi-square (χ^2^) statistic. A 2 × 2 contingency table was used for test of the differences of allele frequencies between cases and controls. The test with additive model and Cochran-Armitage χ^2 ^trend test for the combined equal additive effects were used to identify differences in genotype distribution between cases and controls. Odds ratios (OR) and 95% confidence intervals (CI) were calculated to test the relative risk for association. Statistical powers were calculated using software of PowerSampleSize (PS version 2.1.31). The sample sizes of cases and controls was sufficient to detect association with 80% power (5% level) if allele frequencies 0.15 and 0.20. Tests for association between genotypes and quantitative traits were performed by using ANOVA for normally distributed traits, or alternatively Kruskal-Wallis analysis of ranks for traits with non-normal distributions. Normal probability plots were created and parameter distributions were transformed to natural logarithm when needed to improve the skew-ness and for obtaining a normal distribution before performing statistical analysis of the relevant phenotypes. Analyses were performed using BioMed Data Program (BMDP ver1.12, Cork, Ireland) and Statistica (ver7.1, StatSoft, Tulsa, OK, USA).

## Results

### Single gene analyses

We began with analysis of SNPs rs35069869, rs35368790 and rs7639705 in the *MCF2L2 *gene. Independent segregation of alleles for these studied SNPs were tested in the GoKinD population and they were kept in HWE (P > 0.05). Genetic association analyses with Armitage's trend test, additive and dominant models were performed and data are summarized in Table 2. In SNP rs7639705 (T1165G, Leu359Ile), the G allele frequency in the patients with DN compared to the patients without DN was significantly lower in females (16.5% vs. 22.0%, P = 0.017, OR = 0.701) but not in males (19.7% vs. 22.4%, P = 0.275). Comparison analysis of genotype distribution indicated a significant association between this polymorphism and DN among females (P = 0.018, OR = 0.757). The same analyses in SNPs rs35069869 and rs35368790 between cases (T1DM with DN) and controls (T1DM without DN) were done and no statistically significant association was found.

**Table 2:** Association between the MCF2L2 genetic polymorphisms and diabetic nephropathy

SNP ID Hit Orientation	Group	Genotype Frequency			Allele Frequency		Association Significance P-value/Odds ratio (95% CI)
rs35069869	Women	AA	AG	GG	A	G	
A/G	T1D without DN	174 (0.489)	153 (0.430)	29 (0.081)	0.704	0.211	
	T1D with DN	135 (0.509)	109 (0.411)	21 (0.079)	0.715	0.285	0.661 ^a^
	Men						
	T1D without DN	125 (0.519)	101 (0.419)	15 (0.062)	0.728	0.272	
	T1D with DN	150 (0.479)	137 (0.438)	26 (0.083)	0.698	0.302	0.273 ^a^
rs35368790	Women	GG	GC	CC	G	C	
G/C	T1D without DN	168 (0.471)	151 (0.423)	38 (0.106)	0.682	0.318	
	T1D with DN	134 (0.511)	107 (0.408)	33 (0.105)	0.716	0.284	0.204 ^a^
	Men						
	T1D without DN	107 (0.442)	110 (0.455)	25 (0.103)	0.669	0.331	
	T1D with DN	146 (0.466)	134 (0.428)	33 (0.105)	0.681	0.319	0.696 ^a^
rs7639705	Women	TT	TG	GG	T	G	
T1165G	T1D without DN	213 (0.603)	125 (0.354)	15 (0.043)	0.780	0.220	
Leu359Ile	T1D with DN	188 (0.712)	65 (0.246)	11 (0.042)	0.835	0.165	0.017/0.701 (0.524-0.938) ^a^ 0.018/0.757 ^b^
	Men						
	T1D without DN	157 (0.651)	73 (0.303)	11 (0.046)	0.803	0.197	
	T1D with DN	183 (0.590)	115 (0.371)	12 (0.039)	0.776	0.224	0.275 ^a^

T1D = type 1 diabetes, DN = diabetic nephropathy. Association significances = a. test for allele frequency difference (additive model), and b. for Armitage's trend.

**Figure 1:**
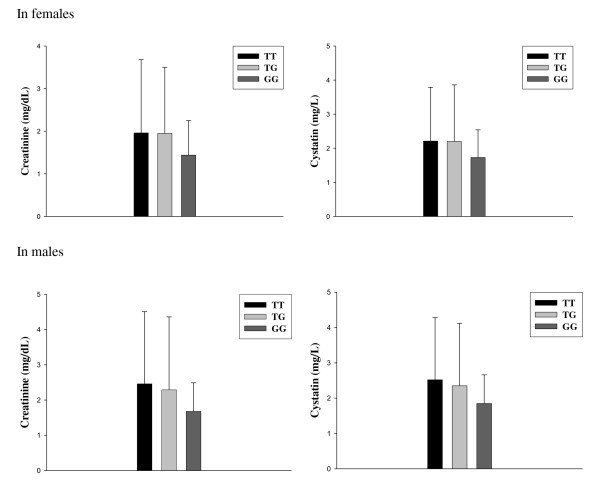
**Variation of creatinine and cystatin levels among female and male type 1 diabetes patients with diabetic nephropathy according to the genotypes of Leu359Ile variant in the ***MCF2L2 ***gene**. Untransformed mean values (± SD) for creatinine and cystatin levels in female and male type 1 diabetes patients with DN are shown. The patients with DN carrying GG genotype had tendency decreased levels of creatinine and cystatin compared the carriers with TT and TG genotypes. But, the differences were not statistically significant mainly due to the higher standard deviations.

We further detected the association between phenotypes and genotypes of SNP rs7639705 (T1165G, Leu359Ile) in female T1DM patients with or without DN. Figure 1 represents variations of the creatinine and cystatin levels in female and male T1DM patients with DN according to the genotypes of SNP rs7639705. In females, the DN patients carrying TT and TG genotypes had similar levels of creatinine (1.96 ± 1.55 and 1.95 ± 1.72 mg/mL) and cystatin (2.20 ± 1.66 and 2.21 ± 1.58 mg/L) in mean values, while the carriers with GG genotype had tendency decreased mean values of creatinine and cystatin levels (1.44 ± 0.81 mg/mL; 1.73 ± 1.09 mg/L) compared to the carriers with TT and TG genotypes. But, the differences were not statistically significant in both creatinine and cystatin levels (P = 0.074 and 0.058) mainly due to the higher standard deviations. Among male T1DM patients with DN, the GG genotype carriers also showed tendency decreased but not significant mean values of creatinine (1.68 ± 0.57 mg/mL vs. 2.29 ± 2.07 and 2.46 ± 2.05 mg/mL) and cystatin (1.85 ± 0.87 mg/L vs. 2.35 ± 1.77 and 2.52 ± 1.76 mg/L) levels compared to the TT and TG genotype carriers. There was no difference in creatinine and cystatin levels among T1DM patients without DN in both females and males who carry three different genotypes (data not shown).

### Multi-gene analyses

We have recently genotyped SNPs rs266729 and rs11915160, respectively, in the *ADIPOQ *and *SOX2 *genes in the same cohort of GoKinD study. Data indicate that these two polymorphisms have an interactive association effect with DN in female T1DM patients [15,16]. In the present study, we employed the GMDR analyses to assess the impact of combinations with the model of *MCF2L2 *(rs7639705)-*SOX2 *(rs11915160)-*ADIPOQ *(rs266729) in 622 female subjects. Table 3 presents the gene-gene interaction and impact of *MCF2L2*, *SOX2 *and *ADIPOQ *genetic polymorphisms in DN among female T1DM patients from GoKinD population. Data were obtained from GMDR analyses without covariate adjustment. There was a significant three-gene model (empirical P-value = 0.001). The model had the cross-validation consistency of 10/10 and testing accuracy of 56.2%. The significance was confirmed by a logistic regression model (P < 0.001). The analyses with two-gene model i.e. *MCF2L2 *together with either *ADIPOQ *or *SOX2 *genetic polymorphisms were also performed, and no significant interaction of *MCF2L2*-*ADIPOQ *or *MCF2L2*-*SOX2 *(P = 0.377, 0.623). The GMDR analysis with the same models studied in females was also performed in male subjects and no significant data were observed.

**Table 3:** MCF2L2, Sox2 and AdipoQ gene-gene interaction and impact on diabetic nephropathy in female type 1 diabetic patients

Best combination	Cross-validation consistency	Testing accuracy (%)	P-value
MCF2L2 (rs7639705) - Sox2 (rs11915160) - AdipoQ (rs266729)	10/10	0.562	0.001
MCF2L2 (rs7639705) - Sox2 (rs11915160)	10/10	0.513	0.623
MCF2L2 (rs7639705) - AdipoQ (rs266729)	10/10	0.545	0.377

P-values were based upon 1000 permutations.

## Discussion

We have used a positional candidate gene research approach to search for the susceptibility or resistance genes in DN. Three candidates including *MCF2L2, ADIPOQ *and *SOX2 *in chromosome 3q26-27 are selected for the genotyping analyses. The *MCF2L2 *gene has the distances of 1,463 kb and 3,415 kb, respectively, to the location of *SOX2 *and *ADIPOQ*. In the present study, we found that SNP rs7639705 (T1165G Leu359Ile) in the *MCF2L2 *gene is significantly associated with decreased risk of DN in female T1DM patients in the GoKinD population. The patients carrying GG genotype have tendency decreased creatinine and cystatin levels compared to the TT and TG carriers. Serum creatinine and cystatin levels are useful for prediction of the impact on renal function measurement and prognostic stages of DN. Therefore, the data suggest that *MCF2L2 *may have resistant genetic effects on DN.

Takeuchi et al. have reported that two intronic polymorphisms i.e. rs35069869 and rs35368790 in the *MCF2L2 *gene are associated with T2DM in a Japanese population [19], but the Leu359Ile polymorphism is not included in their study. Interestingly, *FARP2 *encoded for FERM, pleckstrin domain protein 2 is another Rho GEF in the same family. Savage et al. have demonstrated that rs757978 in the *FARP2 *gene is associated with ESRD in T1DM, and the G allele frequency of this polymorphism in T1DM patients with ESRD was lower than the control subjects (0.090 vs 0.118, P = 0.008) [23]. Rs757978 is a non-synonymous polymorphism causing amino acid change from Thr to Ile at the code 260 of *FARP2 *cDNA. This is similar to what we have found in the *MCF2L2 *gene. Furthermore, the recent biological studies have indicated that GEFs of Rho family may regulate GTPase Cdc42 activity and subsequently contribute to the processes of macro-and/or micro-vascular diseases [24,25]. Therefore, we have a hypothesis that GEFs of Rho family may play an important role in diabetic micro-vascular complications. At the present stage, information of *MCF2L2 *and *FARP2 *in patho-physiology is very limited. Further investigation is necessary to investigate the direct effect of the Rho family GEFs, particularly *MCF2L2 *and *FARP2*, on the development of DN or indirect influence via variation of GTPase activity.

Several genetic linkage analyses have predicted that a chromosomal region on 3q is linked to diabetes and DN [7-13]. Therefore, researchers have searched for the susceptibility and/or resistance genes of DN in the region of this chromosomal arm. He et al. have recently genotyped 3072 tag SNPs) spanning a 28 Mb region around chromosome 3q22 and found that rs1866813 is associated with DN. However, this is an intergenic polymorphism and no gene conferring the susceptibility risk to DN has been identified [26]. Vionnet et al. have selected and studied 14 candidate genes including *AGTR1, PTX3, IL12A, SLC2A2, TNFSF10, ECE2, THPO, EHHADH, HRG, KNG1, AdipoQ, SST, PPP1R2 *and *APOD *from chromosome 3q24-29 and found that the *AdipoQ *promoter polymorphism rs17300539 is associated with DN in T1DM patients in Danish, but not in French and Finnish populations [14]. We have studied the candidate genes including *TRPC1 *(3q22-24), *MME *(3q25), *SOX2 *(3q26.3), *MCF2L2 *(3q27) and *ADIPOQ *(3q27). We have found that the AdipoQ promoter polymorphism-11377C/G is associated with DN in female subjects from the GoKinD population [15]. This polymorphism alters the sequence in one of transcription factor SP1 binding sites and causes reduction of *ADIPOQ *promoter activity [27,28]. Moreover, the investigators in the field of stem cell research have amply demonstrated that four transcription factors, including *Oct3/4, Sox2, Klf4 *and *c-Myc*, play an important role in induction of pluripotent stem cells from somatic cells ([29-32]. We have recently performed a genetic study of the *Sox2*gene in the same GoKinD cohort as studied in the *MCF2L2 *and *AdipoQ *genes, and found that *Sox2 *genetic polymorphism is associated with DN in female T1DM subjects [16]. The MCF2L2 gene has the distances of 146 kb and 341 kb, respectively, to the location of *Sox2 *and *AdipoQ*. In order to evaluate whether *MCF2L2 *has gene-gene interaction with *ADIPOQ *and *Sox2 *in term of association with DN, we have performed gene-gene interaction analysis. The results suggest that *MCF2L2 *has combined genetic effects with both *ADIPOQ *and *Sox2 *on DN among female subjects but not with either *ADIPOQ *or *Sox2 *alone. The linkage of chromosome 3q to DN is most likely determined by the multiplex loci, including these three genes.

Evidence has indicated that gender is an important risk factor for the development and progression of DN. This disease appears less frequently in females than in males with a ratio of approximately 1:1.3 [33]. Previously, Pettersson-Fernholm et al. have demonstrated that the *AT2 *genetic polymorphisms confer risk susceptibility to the development of DN in male but not female T1DM subjects of Finnish population [34]. In the present study, we have demonstrated that *MCF2L2*, *AdipoQ *and *Sox2 *genetic polymorphisms have protective effects on DN in female T1DM patients. The genetic association of *MCF2L2*, *AdipoQ *and *Sox2 *genetic polymorphisms with DN in male T1DM patients is not observed. The pattern of tendency decreased creatinine and cystatin levels in DN patients carrying GG genotype compared to the TT and TG carriers is seen in both females and males. Therefore, the genotypic and phenotypic differences in gender should be taken into our consideration in genetic and functional analyses of DN.

## Conclusions

The present study provides the first evidence that the *MCF2L2 *Leu359Ile polymorphism is associated with decreased risk of DN in female T1DM patients. *MCF2L2 *together with *ADIPOQ *and *Sox2 *genetic polymorphisms have combined genetic effects on DN in females, which reveals that the linkage of chromosome 3q to DN may be explained by the accumulated effects of multi-genes.

## Competing interests

The authors declare that they have no competing interests.

## Authors' contributions

DZ performed genotyping experiments and data analysis. SE, KB and HFG conceived of the study and also evaluated the data. HFG participated in experiments and data analysis. All authors prepared and approved the manuscript.

## Pre-publication history

The pre-publication history for this paper can be accessed here:

http://www.biomedcentral.com/1471-2350/11/116/prepub
